# A rapid RT-LAMP SARS-CoV-2 screening assay for collapsing asymptomatic COVID-19 transmission

**DOI:** 10.1371/journal.pone.0273912

**Published:** 2022-09-01

**Authors:** Rebecca C. Allsopp, Caroline M. Cowley, Ruth C. Barber, Carolyn Jones, Christopher W. Holmes, Paul W. Bird, Shailesh G. Gohil, Claire Blackmore, Martin D. Tobin, Nigel Brunskill, Philip N. Baker, Jacqui A. Shaw

**Affiliations:** 1 Leicester Cancer Research Centre, Department of Genetics and Genome Biology, University of Leicester, Leicester, United Kingdom; 2 Leicester Molecular Diagnostics, Leicester Cancer Research Centre, University of Leicester, Leicester, United Kingdom; 3 Leicester Precision Medicine Institute, University of Leicester, Leicester, United Kingdom; 4 Clinical Microbiology, University Hospitals of Leicester NHS Trust, Leicester Royal Infirmary, Leicester, United Kingdom; 5 Department of Respiratory Sciences, University of Leicester, Leicester, United Kingdom; 6 Centre for Environmental Health and Sustainability, University of Leicester, Leicester, United Kingdom; 7 Department of Health Sciences, University of Leicester, Leicester, United Kingdom; 8 Leicester NIHR Biomedical Research Centre, Leicester, United Kingdom; 9 Department of Cardiovascular Sciences, University of Leicester, Leicester, United Kingdom; 10 College of Life Sciences, University of Leicester, Leicester, United Kingdom; University of Helsinki: Helsingin Yliopisto, FINLAND

## Abstract

**Purpose:**

To demonstrate the diagnostic performance of rapid SARS-CoV-2 RT-LAMP assays, comparing the performance of genomic versus sub-genomic sequence target with subsequent application in an asymptomatic screening population.

**Methods:**

RT-LAMP diagnostic specificity (DSe) and sensitivity (DSe) was determined using 114 RT-PCR clinically positive and 88 RT-PCR clinically negative swab samples processed through the diagnostic RT-PCR service within the University Hospitals of Leicester NHS Trust. A swab-based RT-LAMP SARS-CoV-2 screening programme was subsequently made available to all staff and students at the University of Leicester (Autumn 2020), implemented to ISO 15189:2012 standards using NHS IT infrastructure and supported by University Hospital Leicester via confirmatory NHS diagnostic laboratory testing of RT-LAMP ‘positive’ samples.

**Results:**

Validation samples reporting a Ct < 20 were detected at 100% DSe and DSp, reducing to 95% DSe (100% DSp) for all samples reporting a Ct < 30 (both genomic dual sub-genomic assays). Advisory screening identified nine positive cases in 1680 symptom free individuals (equivalent to 540 cases per 100,000) with results reported back to participants and feed into national statistics within 48 hours.

**Conclusion:**

This work demonstrates the utility of a rapid RT-LAMP assay for collapsing transmission of SARS-CoV-2 in an asymptomatic screening population.

## Background

According to Government guidance published by the Medicines and Healthcare Products Regulations Agency (Target Product Profile for Laboratory-Based SARS-CoV-2 Viral Detection tests), a dual (or more) target SARS-CoV-2 RNA format is desirable for diagnostic testing, but use of a single target is acceptable. Dual target assays protect against false-negative results caused by genome sequence mutations in the assay target sites and can offer improved certainty in results when the results of both targets are in agreement, but interpretation of results can be complicated where the results are discrepant. The interpretation and assessment of clinical significance of positive SARS-CoV-2 RT-PCR results can be equally challenging (PHE guide ‘Understanding cycle threshold’ (Ct) in SARS-CoV-2 RT-PCR’). A positive result with low viral load (high Ct) can be seen in both the early stages of infection (before the person becomes capable of transmission) or later in infection when transmission risk is low [1]. If therefore, the purpose of a public health utility test is to identify individuals who are currently infectious then data from highly sensitive RT-PCR needs careful interpretation.

Mass demand and delays associated with centralised RT-PCR testing and reagent availability were soon recognised as major obstacles in effectively responding to the SARS-CoV-2 pandemic with mitigation strategies urgently required [2]. Use of an alternative rapid and cheaper isothermal reverse transcriptase loop mediated amplification (RT-LAMP) strategy [3] with its completely different design, equipment and reagent requirements could largely bypass some of the challenges detailed above. Reactions comprise of 4–6 primers targeting 6–8 template region, typically spanning in excess of 150 bp. Therefore, detection of slowly degrading RNA fragments, of historical no longer contagious infection, is less likely with RT-LAMP than with conventional RT-PCR designed to amplify significantly shorter sequence regions.

Fast amplification via LAMP can be detected by a variety of endpoint readouts including fluorescence, turbidity and colorimetric change optimal for point of care LAMP-based diagnostics [4]. Several groups demonstrated the suitability of an RT-LAMP detection strategy early on in the pandemic. Rabe and Cepko [5] optimised primers directed toward a non-conserved region of the SARS-CoV-2 Orf1a gene, (assay termed Orf1a-Harvard Medical School enhanced or ‘Orf1a-HMSe’). New England Biolabs (NEB) developed a dual sub-genomic assay targeting regions of the N and E gene, plus a separate internal control assay targeting the human beta actin gene (ACTB) for confirmation of total RNA indicative of appropriate sample collection [6]. Marino et al., [7] went on to multiplex the Orf1a-HMSe, N and E primer sets (plus a primer set targeting 18S RNA), developing an extraction-less Prime CovidDetect^™^ Rapid Detection kit. Fowler et al., [8] optimised and validated OptiGene’s COVID-19 RT-LAMP workflow, successfully establishing the first CE-IVD registered RT-LAMP kit, implemented nationally across several NHS Trusts.

The work presented herein compares the choice of SARS-CoV-2 RT-LAMP targets (genomic versus sub-genomic) and end-point readouts (colorimetric versus fluorescent detection). Assay limit of detection was confirmed using synthetic SARS-CoV-2 positive control RNA of known concentration, and evaluation of diagnostic sensitivity (DSe) and specificity (DSp) was confirmed using residual RNA from UHL NHS inpatient oropharyngeal / nasopharyngeal (ON) swab samples with corresponding RT-PCR Ct value. A swab based fluorescent end-point rapid RNA to RT-LAMP reaction targeting the genomic Orf1a with parallel total RNA internal control reaction to mitigate reporting of false negative results subsequently provided the basis of an asymptomatic screening programme available to all staff and students at the University of Leicester.

## Methods

### Ethics statement

The ON swab samples used in this study were collected in the context of routine clinical patient care and the RT-LAMP analyses reported herein are reporting the outcome of service development work undertaken in order to increase diagnostic test capacity, rather than as research. The assays were performed on residual de-identified patient material in a UKAS-accredited diagnostic service laboratory supporting diagnostic processes including RNA extractions for the standard RT-PCR assay; thereby not requiring informed consent and ethics committee approval.

### University of Leicester asymptomatic screening programme

**Fig 1:**
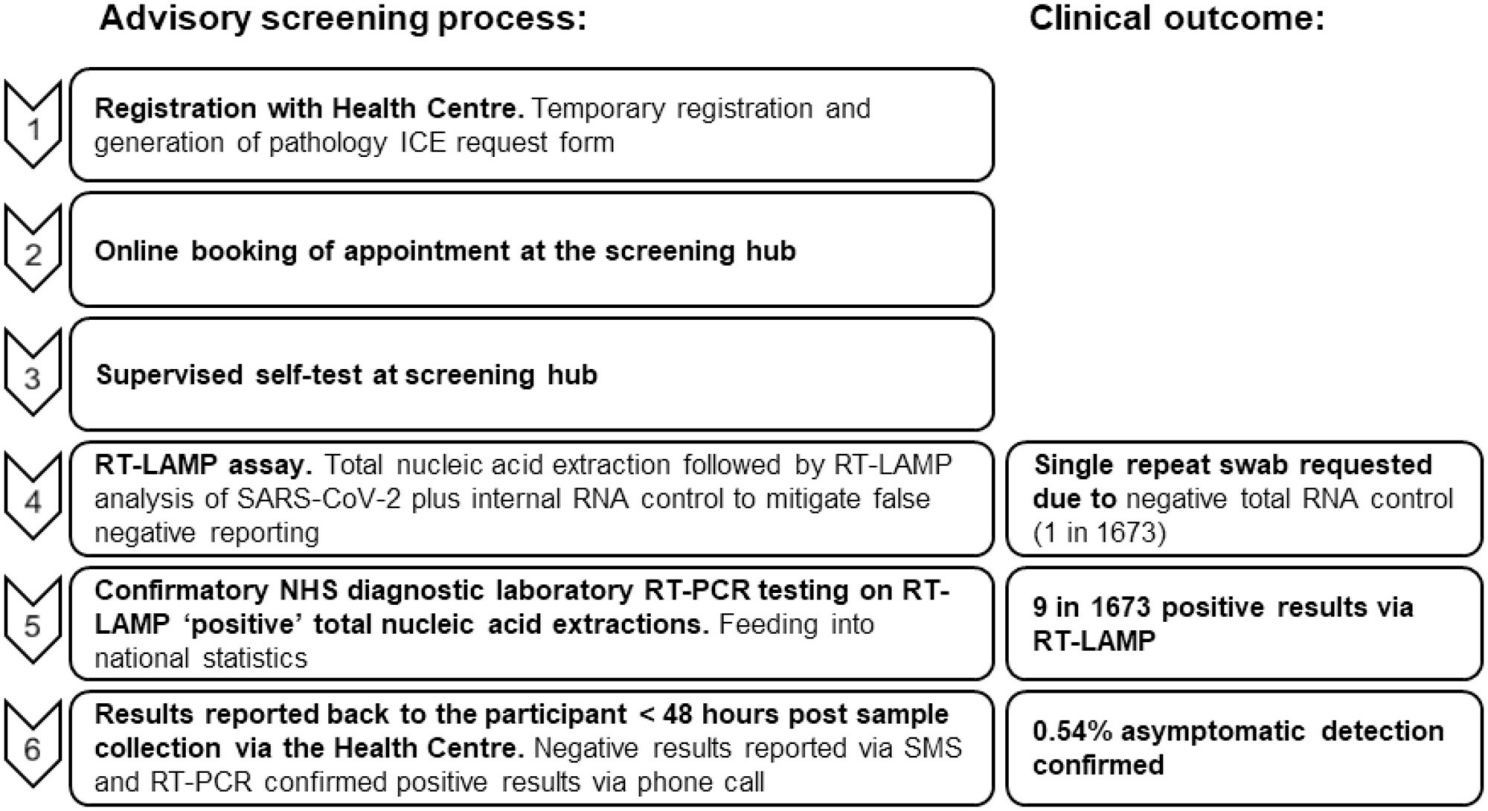
University of Leicester SARS-CoV-2 advisory screening programme. Available to all students and staff without symptoms for a period of twelve weeks (October 2020 –December 2020) to allow rapid isolation and reduce outbreaks.

Participants accessing the screening programme required registration at the primary care Leicester Victoria Park Health Centre for generation of a pathology request form. Individuals self-swabbed (throat and lower nasal cavity) at a supervised screening venue using Miraclean swabs placed into PrimeStore Molecular Transport Medium for viral inactivation and RNA stabilisation at room temperature (Longhorn Vaccines and Diagnostics). Within a 24-hour period, total nucleic acid extraction was followed by RT-LAMP targeting the Orf1a plus an internal total RNA control reaction. Data was uploaded to the pathology iLab system and NHS laboratory RT-PCR testing confirmed any positive RT-LAMP results, feeding into the national track and trace system. Results were reported back to participants (< 48 hours post sample collection) via SMS (negative result) or phone call (positive results) from the Victoria Park Health Centre. Fig 1 details the programme workflow.

### Control RNA

Synthetic SARS-CoV-2 RNA at a concentration of 1×10^6^ RNA copies per microliter was purchased from Twist Bioscience and diluted appropriately in nuclease free water (Twist Synthetic SARS-CoV-2 RNA Control 1 [MT007544.1]—SKU: 102091 and Control 2 [MN908947.3]—SKU: 102024). Negative control RNA from related Betacoronavirus 1 (Strain OC43) and non-related Influenza A (H1N1) was purchased from ATCC. Total human RNA purchased from Invitrogen (4307281).

### Swab sample collection and RNA extraction of RT-LAMP assay validation material

Standard ON swabs from hospital inpatients were collected using PHE-approved flocked swabs placed into viral transport media (Virocult / VTM-M4RT). RNA extraction (on 200 μl of inactivated sample mixed with 265 μl Binding Solution) was carried out using the MagMAX Viral/Pathogen Nucleic Acid Isolation kit (MVPII, ThermoFisher) on the KingFisher Flex Purification System. Residual patient RNA samples used for validation purposes potentially suffered a degree of sample degradation during prolonged storage prior to RT-LAMP.

### NHS real-time RT-PCR

Purified nucleic acid from ON swab samples was reverse transcribed into cDNA and amplified using a CE marked, locally validated commercially available kit targeting the E and S-gene sequence regions (RealStar^®^ SARS-CoV-2 RT-PCR kit, Altona Diagnostics GmbH, Hamburg, Germany). Samples were considered positive for SARS-CV-2 is either the S gene or E gene report a Ct < 40.

### RT-LAMP primers

Primers (HPLC grade, Merck) targeting the Orf1a (Orf1a-HMSe) were designed by Rabe and Cepko [5]. Primers targeting the nucleocapsid (N), envelope gene (E) and internal human beta-actin internal control (ACTB) were designed by NEB [6]. All primer sequences are listed in S1 Table. Individual RT-LAMP primer sets were prepared as 20 times final concentration stock, final assay concentrations of 0.2 μM F3/B3, 1.6 μM FIP/BIP and 0.4 μM LoopF/Loop B.

### Fluorescent RT-LAMP

Reactions contained 1 X WarmStart^®^ LAMP Master Mix (E1700) supplemented with 1 X fluorescent dye (NEB dye provided with E1700 master mix), 0.02 U/μL Antarctic Thermolabile UDG (NEB), 700 μM dUTP (NEB) and 1 X standard concentration LAMP primers. Assay validation reactions were prepared to a final reaction volume of 25 μl with nuclease free water and incubated at 65°C for 40 minutes using the StepOnePlus (Applied Biosystems) or Rotor-Gene Q (Qiagen) thermoclycler. For the screening programme, RNA extracted from participants samples were run on the Rotor-Gene Q using the SARS-CoV-2 Or1a assay (plus ACTB control) for 20 minutes, equivalent to a Ct <30.

### Colorimetric RT-LAMP

Reactions contained 1 X WarmStart^®^ Colorimetric LAMP Master Mix (M1800; NEB) supplemented with 1 x EvaGreen (Biotium), 0.02 U/μL Antarctic Thermolabile UDG (NEB), 700 μM dUTP (NEB), 1 X standard concentration LAMP primers and 40 mM guanidine chloride solution (Sigma G3272, pH adjusted to pH ~8). Reactions were prepared to final volume of 25 μl using nuclease free water, incubated at 65°C for 40 minutes on a StepOnePlus thermocycler (Applied Biosystems). The colour of finished reactions was recorded using an office flatbed scanner.

### Statistical analysis

Time to positive (TTP in minutes) served as a surrogate for RT-PCR Ct and a semi-quantitative measure of viral RNA concentration. Additional product specificity checks were provided by melt curve analysis within acceptance range (2 degrees either side of the mean Tm determined during assay validation using patient samples). Validation data using synthetic material represent the average of two independent experiments, performed in quadruplicate, presented as mean TTP ± S.E.M. Validation data using residual patient RNA represent a single reaction performed in parallel reactions targeting the Orf1a, N+E and ACTB total RNA control sequence. MedCalc^®^ Scientific Software was used for diagnostic test evaluation to determine the diagnostic sensitivity (DSe) referring to the proportion of known positive samples that tested positive in the assay and diagnostic specificity (DSp) referring to the proportion of samples from known negative reference samples that test negative in the assay. Analyses was grouped according to RT-PCR threshold, whereby either the S or the E gene reported a Ct of <20, <30 and <40.

## Results

### Genomic versus sub-genomic RT-LAMP assay target validation

**Fig 2:**
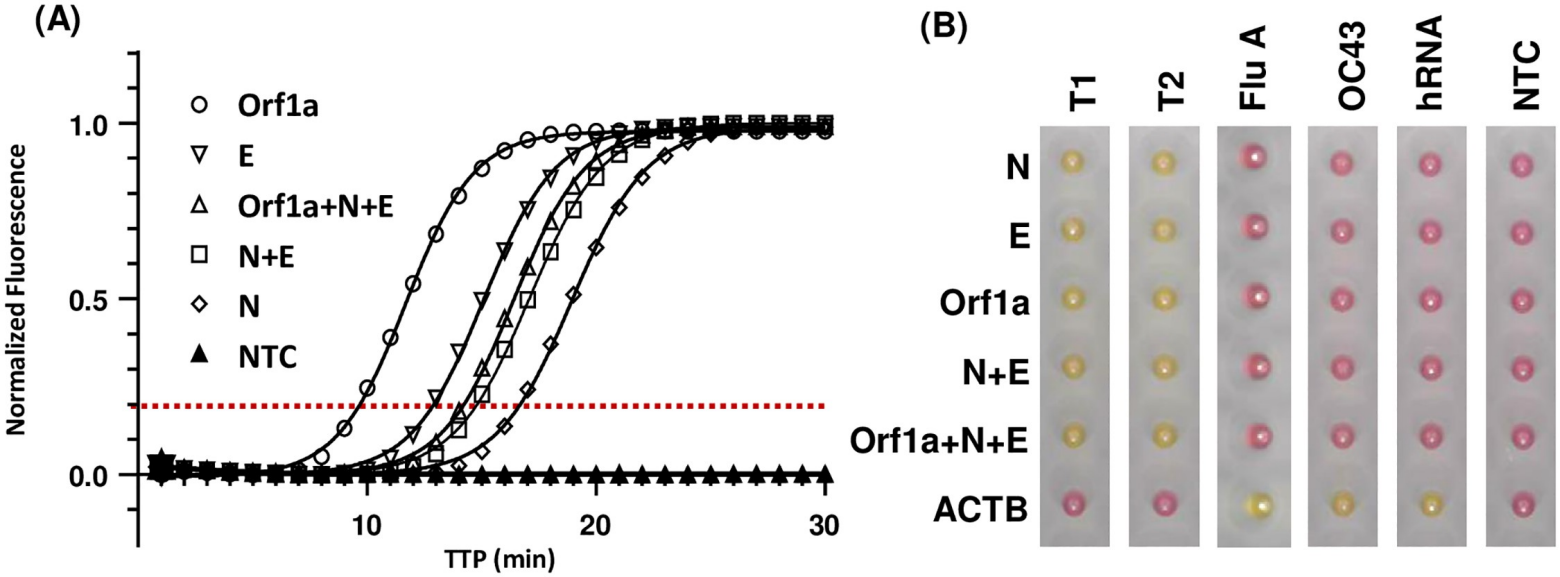
RT-LAMP primer investigation. SARS-CoV-2 RNA primer sets targeting the nucleopcapsid (N), envelope gene (E) (NEB design) and Orf1a (Rabe and Cepko Harvard Medical School) were tested independently and in combination (dual N+E reaction and multiplex Orf1a+N+E reaction) against 1x10^4^ copies of Twist synthetic SARS-CoV-2 control RNA (T1 and T2). Negative control RNA from Betacoronavirus 1 strain OC43 and Influenza A (H1N1) at a single concentration (1x10^5^ copies per well) plus a water no template control (NTC). A total RNA control primer set (NEB) targeting human beta actin (ACTB) was also included and tested against 5 ng total human RNA (hRNA). Both RT-LAMP fluorescent end-point and colorimetric 25 μl reactions were performed at 65°C for 40 minutes on a StepOnePlus thermoclycler. (A) Representative fluorescent amplification curves where time to positive (TTP) is the time at which amplification exceeds the manually set, reaction consistent threshold (red dotted line). (B) Representative colorimetric reactions whereby yellow indicates positive amplification and pink no amplification.

The performance of fluorescent and colorimetric end-point RT-LAMP reactions targeting the genomic Orf1a and sub-genomic N and E gene regions were tested solo, in duplex (N+E) and in multiplex combination (Orf1a+N+E) against a single concentration of two synthetic positive control RNAs (Fig 2). Fluorescent end-point data presented in Fig 2A demonstrates equivalent amplification of the Twist positive controls (T1 and T2) with no statistically significant difference. Reactions targeting the Orf1a were fastest to exceed the threshold (9.1 ± 0.07 and 8.7 ± 0.16, T1 and T2 respectively), followed by reactions targeting the E gene (12.1 ± 0.09 and 12.4 ± 0.10 minutes) and finally the N gene (17 ± 0.17 and 16.7 ± 0.17 minutes). Dual N+E reactions exceeded the amplification threshold at a mid-point between N and E alone (14.3 ± 0.10 and 14.2 ± 0.08 minutes). Finally, addition of the Orf1a primer set to the dual N+E reaction further enhanced dual velocity (13.4 ± 0.22 and 13.0 ± 0.21 minutes). A lack of cross-reactivity of all SARS-CoV-2 targets was confirmed by a lack of amplification in negative control wells (related human coronavirus OC43, non-related influenza A and total human RNA). Presence of viable RNA within these samples was confirmed by control ACTB amplification (8.9 ± 0.25, 13.0 ± 0.19 and 9.5 ± 0.09 respectively). The ACTB total RNA control reaction failed to amplify the positive RNA controls consistent with these being synthetic material. Finally, no template controls (NTC) confirmed the absence of non-specific amplification in any reactions. Fluorescent data is summarised in Table 1. Summary melt curve data was also collated for each primer set (Orf1a 82.1 ± 0.058, N+E 89.4 ± 0.060, ACTB 92.7 ± 0.050), and used as an amplification product specificity check in subsequent analyses of patient samples. End-point colorimetric detection (Fig 2B) consistent with fluorescent findings, demonstrated clear amplification in under 30 minutes with a colour change from pink to yellow for all primers (Orf1a, N and E) and primer combinations (N+E & Orf1a+N+E) against positive RNA controls T1 and T2. SARS-CoV-2 targeting reactions failed to amplify negative controls (OC43, influenza A and total human RNA), whilst the ACTB internal control reaction confirmed the presence of viable template RNA. ACTB internal control reaction failed to amplify synthetic material (as expected) and a NTC confirmed the absence of non-specific amplification in any reactions.

**Table 1:** RT-LAMP primer investigation.

SARS-CoV-2 target:	TTP (minutes):					
	Twist 1 (SKU 102019)	Twist 2 (SKU 102024)	OC43	Influenza A	Human RNA	NTC
**Orf1a**	9.1 ± 0.07	8.7 ± 0.16	na	na	na	na
**N**	17 ± 0.17	16.7 ± 0.17	na	na	na	na
**E**	12.1 ± 0.09	12.4 ± 0.10	na	na	na	na
**N+E**	14.3 ± 0.10	14.2 ± 0.08	na	na	na	na
**Orf1a+N+E**	13.4 ± 0.22	13.0 ± 0.21	na	na	na	na
**ACTB**	na	na	13.0 ± 0.19	9.5 ± 0.09	8.9 ± 0.25	na

Data summary of an average of 2 independent experiments each performed in quadruplicate, presented as mean TTP ± S.E.M. No amplification is noted as ‘na’.

### Limit of detection of fluorescent and colorimetric end-point RT-LAMP assays

**Fig 3:**
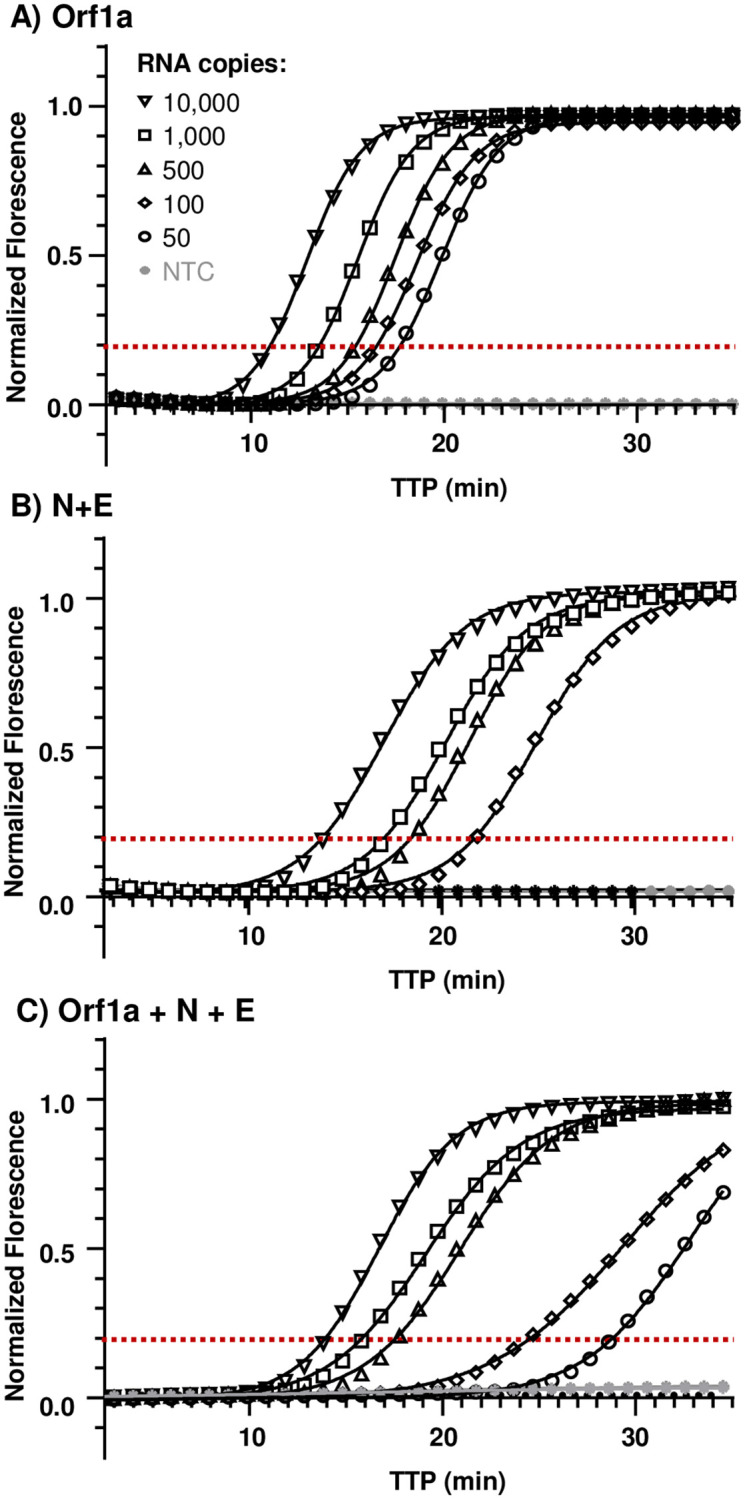
Limit of detection of fluorescent end-point RT-LAMP reactions targeting genomic and sub-genomic regions of SARS-CoV-2. Twist Bioscience synthetic positive control RNA (control 2 GenBank ID MN908947.3, GISAID Wuhan-Hu-1) was serially diluted to 10,000, 1,000, 500, 100, 50 and 10 copies of viral sequence per 25 μl reaction. Water no template control (NTC) were included in each reaction. Reactions were performed at 65°C for 40 minutes on the Qiagen Rotor-Gene Q Thermoclycler platform. Representative amplification and linear regression analysis for each primer set are shown. (A) RT-LAMP targeting the Orf1a. (B) RT-LAMP targeting N+E duplex. (C) RT-LAMP triple Orf1a+N+E target. Time to positive (TTP) is the time at which amplification exceeds the manually set, reaction consistent threshold (red dotted line) when amplification enters the rapid linear, exponential phase. Data represents the average of two experiments each performed in quadruplicate.

We assessed the limit of detection of fluorescent and colorimetric end-point RT-LAMP reactions targeting the Orf1a, dual N+E gene and multiplex Orf1a+N+E genes of SARS-CoV-2. Parallel fluorescent and colorimetric end-point reactions were performed against synthetic RNA (Twist control 2) serially diluted to 10,000, 1,000, 500, 100, 50 and 10 copies of viral sequence. Representative fluorescent end-point amplification curves and linear regression analysis of primer sets Orf1a, N+E duplex and multiplex Orf1a+N+E target are presented in Fig 3 with summary data presented in Table 2. RT-LAMP targeting Orf1a was the fastest to exceed amplification threshold at 8.9 ± 0.12 minutes (10,000 viral RNA copies), capable of reproducibly detecting 500 copies synthetic viral RNA. Lower viral loads down to 10 copies were detectable within 20 minutes although not reproducibly. RT-LAMP dual targeting N+E and multiplex Orf1a+N+E were ~5 minutes slower to exceed amplification threshold for equivalent viral loads, analogously capable of reproducibly detecting 500 copies of synthetic viral RNA. Colorimetric reactions (S2 Table) augmented with EvaGreen intercalating dye and guanidine hydrochloride were equally able to reproducibly detect 500 viral RNA copies, exceeding the amplification threshold slightly faster that their fluorescent counterpart (2.7, 5.6 and 4.5 minutes faster for Orf1a, N+E and Orf1a+N+E respectively for 500 viral copies). All reactions that exceeded the amplification threshold (indicated by intercalating dye associated TTP) also showed a clear visual colorimetric change from pink to yellow in under 30 minutes.

**Table 2:** Limit of detection of fluorescent end-point RT-LAMP reactions.

Synthetic positive control RNA	Fluorescent RT-LAMP Mean TTP (min) ± SEM (N)		
Copies / reaction	Orf1a	N+E	Orf1a+N+E
**10,000**	8.9 ± 0.12 (8/8)	14.4 ± 0.16 (8/8)	14.1 ± 0.12 (8/8)
**1,000**	11.6 ± 0.49 (8/8)	17.1 ± 0.77 (7/8)	17.2 ± 0.50 (8/8)
**500**	13.3 ± 0.65 (8/8)	21.4 ± 2.51 (8/8)	17.2 ± 0.29 (8/8)
**100**	14.1 ± 0.0 (1/8)	22.0 ± 1.44 (5/8)	23.2 ± 1.66 (5/8)
**50**	16.4 ± 3.91 (3/8)	na	29.2 ± 0.0 (1/8)
**10**	11.6 ± 0.0 (1/8)	na	21.1 ± 0.85 (2/8)
**NTC**	na	na	na

Reactions targeting the Orf1a, dual N+E gene and multiplex Orf1a+N+E of SARS-CoV-2 using a serially diluted Twist Bioscience synthetic positive control RNA. Data summary is an average of 2 independent experiments, performed in quadruplicate and presented as mean TTP ± S.E.M. Numeration in parentheses indicates the number of repeat reactions achieving the amplification threshold required to report a TTP. No amplification (‘na’).

### Diagnostic validation of fluorescent end-point RT-LAMP assays

Residual RNA extracted from patient ON swab samples originally processed through the diagnostic RT-PCR service in the UHL Trust were processed through parallel RT-LAMP reactions targeting the Orf1a, dual N+E gene and ACTB internal control. Two PCR platforms were assessed: the ABI StepOnePlus PCR platform (88 UHL negative and 114 UHL positive samples; S3 Table) and the Qiagen Rotor-Gene Q (40 UHL negative and 38 UHL positive samples; S4 Table). RT-LAMP reactions performed on the StepOnePlus platform with patient samples reporting an original RT-PCR of Ct < 20 were equally detected by Orf1a and N+E RT-LAMP at 100% / 100% (DSe / DSp). DSe / DSp decreased slightly to 91.7% / 100% and 92.7% / 100% for the Orf1a and N+E targets respectively for patient samples reporting an RT-PCR Ct < 30 and then to 79.8% / 100% and 81.6% / 100% DSe / DSp respectively upon processing all patient samples reporting an RT-PCR Ct < 40 (Table 3A). For the Rotor-Gene Q platform, RT-LAMP reactions of samples reporting an original RT-PCR of Ct < 20 were detected equivalently by Orf1a and dual N+E RT-LAMP, reporting 100% / 100% DSe / DSp respectively. This decreased to 94.1% / 100% DSe / DSp for samples reporting an RT-PCR Ct < 30 and finally to 81.6% / 100% and 84.2% / 100% DSe / DSp for samples reporting an RT-PCR Ct < 40 (Table 3B). Additional product specificity check provided by the melt curve (Tm) confirmed selective amplification of the product.

**Table 3:** DSe and DSp of single genomic versus dual sub-genomic SARS-CoV-2 fluorescent end-point RT-LAMP assays performed using residual RNA extracted from clinical patient ON swab samples with comparator RT-PCR Ct values. Assays were run at 65°C for 40 minutes on **A)** StepOnePlus PCR platform (114 UHL positive samples & 88 UHL negative samples) and **B)** Rotor-Gene Q Qiagen PCR platform (38 UHL positive samples & 40 UHL negative samples). Sensitivity and specificity with 95% CI are shown for samples with a corresponding RT-PCR Ct < 20, Ct < 30 and Ct < 40. Calculations performed using the MedCalc Scientific Software.

**A) StepOnePlus; Applied Biosystems**		
**RT-LAMP target:**	**SARS-CoV-2 Orf1a**	**SARS-CoV-2 Dual N+E**
DSe (95% CI)	100.0% (91.6% to 100.0%)	100.0% (91.6% to 100.0%)
DSp (95% CI) **Ct < 20** (N44)	100.0% (95.9% to 100.0%)	100.0% (95.9% to 100.0%)
DSe (95% CI)	91.7% (84.2% to 96.3%)	92.7 (85.6 to 97.0%)
DSp (95% CI) **Ct < 30** (N94)	100.0% (95.9% to 100.0%)	100.0% (95.9 to 100.0%)
DSe (95% CI)	79.8% (71.3% to 86.7%)	81.6% (73.2 to 88.2%)
DSp (95% CI) **Ct < 40** (N114)	100.0% (95.9% to 100.0%)	100.0% (95.9 to 100.0%)
**B) Rotor-Gene Q; Qiagen**		
**RT-LAMP target:**	**SARS-CoV-2 Orf1a**	**SARS-CoV-2 Dual N+E**
DSe (95% CI) **Ct < 20** (N13)	100.0% (75.3% to 100.0%)	100.0% (75.3% to 100.0%)
DSp (95% CI)	100.0% (91.2% to 100.0%)	100.0% (91.2% to 100.0%)
DSe (95% CI) **Ct < 30** (N34)	94.1% (80.3% to 99.3%)	94.1% (80.3% to 99.3%)
DSp (95% CI)	100.0% (91.2% to 100.00%)	100.0% (91.2% to 100.00%)
DSe (95% CI) **Ct < 40** (N38)	81.6% (65.7% to 92.3%)	84.2% (68.8% to 94.0%)
DSp (95% CI)	100.0% (91.2% to 100.0%)	100.0% (91.2% to 100.0%)

### Diagnostic validation of colorimetric end-point RT-LAMP assays

Residual RNA extracted from patient ON swab samples were also processed through colorimetric end-point RT-LAMP reactions targeting the Orf1a, dual N+E gene plus ACTB internal control reaction. Samples reporting an original RT-PCR of Ct < 30 were detected equally by Orf1a and N+E RT-LAMP assays (17 of 19 RT-PCR positive samples and 40 of 40 negative samples), demonstrating 89.5% DSe and 100% DSp. Assay performance decreased to 81.0% / 100% DSe / DSp for patient samples reporting an RT-PCR Ct < 40 (19 of 21 RT-PCR positive samples) (Table 4, S5 Table). Additional performance of a triple target (Orf1a+N+E) assay demonstrated 100% DSe and DSp for samples reporting an RT-PCR Ct < 30 decreasing to 92.70% / 100% DSe / DSp for samples reporting an RT-PCR Ct < 40 (37 of 41 RT-PCR positive samples) (Table 4, S6 Table).

**Table 4:** DSe and DSp of single genomic, dual sub-genomic and triple genomic / sub-genomic SARS-CoV-2 colorimetric end-point RT-LAMP assays.

RT-LAMP target:	SARS-CoV-2 Orf1a	SARS-CoV-2 Dual N+E	SARS-CoV-2 Orf1a+N+E
CT<30 DSe (95% CI)	89.5% (66.7% to 98.7%)	89.5% (66.7% to 98.7%)	100% (89.7% to 100%)
DSp (95% CI)	100% (91.2% to 10%)	100% (91.2% to 100%)	100% (91.2% to 100%)
Ct<40 DSe (95% CI)	81.0% (58.1% to 94.6%)	81.0% (58.1% to 94.6%)	92.7% (80.1% to 98.5%)
DSp (95% CI)	100.0% (91.2% to 100.0%)	100.0% (91.2% to 100.0%)	100.0% (91.2% to 100.0%)

### University of Leicester RT-LAMP asymptomatic screening programme

Screening was performed to ISO 15189:2012 standards, guided by the Leicester Molecular Diagnostic Lab. RNA extracted from throat and lower nasal cavity swabs from 1,673 symptom free individuals attending campus (autumn 2020) were processed through RT-LAMP targeting the Orf1a and internal control ACTB. The RT-LAMP assay was run for 20 minutes, detecting equivalent to a Ct <30 (S4 Table). During this period, a single repeat swab was requested due to inefficient sampling as identified by failure of amplification within the ACTB control reaction indicating absence of total RNA. In total, 9 RT-PCR confirmed RT-LAMP positive results from a total of 1,673 tests demonstrated a prevalence of asymptomatic infection of 0.54% (540 cases per 100,000) demonstrating the value and usability of RT-LAMP molecular diagnostic tool for the detection of SARS-CoV-2 in an asymptomatic population.

## Conclusions

This report demonstrates selective amplification of SARS-CoV-2 viral RNA by rapid and cheap RT-LAMP assays targeting genomic (Orf1a) and dual sub-genomic (N+E) RNA sequence regions via fluorescent or colorimetric determination. Primer sets match the SARS-CoV2 Ref Seq reference genome 100% (Wuhan-Hu-1; NC 045512.1). *In-Silico* inclusivity analysis by Marino et al., [7] demonstrated primer sets to differ by one or less mutation in 99.8%, 99.8% and 99.6% (Orf1, E, and N gene respectively against 5773195 SARS-CoV-2 GISAID deposited sequences), with extremely low potential for poor primer hybridization to occur across all three primer sets. Furthermore, minimal *In-Silico* and wet testing cross-reactivity was observed for pathogens similar or related to SARS [7].

Equivalent sensitivity was observed for genomic (Orf1a) and dual sub-genomic (N+E) targets with assays capable of reproducibly detecting 500 copies of Twist Bioscience synthetic positive control RNA. RT-LAMP targeting the Orf1a was significantly faster to exceed amplification threshold aided by inclusion of a poly T linker within the FIP and BIP primer pairs, facilitating faster loop formation [5]. Inclusion of guanidine chloride within colorimetric reactions also slightly enhanced amplification velocity compared to the fluorescent end-point counterparts [6].

Diagnostic validation of SARS-CoV-2 RT-LAMP reactions using RNA extracted from hospital inpatient ON swabs, demonstrated equivalent DSe and DSp (100% / 100%) for the genomic and dual sub-genomic target assays, concordant with comparator RT-PCR for Ct < 20. A small, comparative drop in DSe across different PCR platforms was observed when including all samples reporting an RT-PCR Ct < 30 (ranging from 91.7% to 94.1%). RT-LAMP was not able to detect RT-PCR confirmed positive samples with a Ct > 33 (the Altona RT-PCR assay reporting positive samples to a CT of under 40). Therefore, upon inclusion of all samples reporting a comparator RT-PCR Ct < 40, the DSe of the Orf1a and N+E RT-LAMP assays performed on the Rotor-Gene Q PCR platform, decreased to between 81.6% and 84.2% (100% DSp). Assays performed on the StepOnePlus platform decreased further to around 80% DSe (100% DSp). Interestingly, the colorimetric triple target (Orf1a+N+E) RT-LAMP performed on the StepOnePlus platform maintained higher sensitivity, reporting DSe / DSp of 92.7% / 100% for all patient samples with Ct < 40. Equivalent triple target fluorescent end-point assays were not assessed due to lack of template RNA.

Ultimately, the superior sensitivity harnessed by RT-PCR presents a well-documented drawback ubiquitous for inferring infectiousness from RT-PCR detection, with slowly degrading SARS-CoV, MERS, Influenza, Ebola and Zika viral RNA all detected long after the disappearance of the infectious virus [9]. An RT-PCR positive therefore reflects an assay’s ability to detect viral RNA and not necessarily the presence of viable virus. The only robust way to detect viable virus is by cell culture, however this method is labour-intensive, slow and not amenable to high-throughput processing so is not suited to large-scale diagnostics. In comparison to shorter amplicon RT-PCR, RT-LAMP assays are designed over a larger RNA template (dictated by the 6-primer annealing locations) thus reducing the likelihood of detecting residual fragments of viral RNA. Pertinently, the anonymised patient swab samples used to validate this study were not paired with clinical data detailing the duration from onset of symptoms, however, patients present to hospital a median of 7 to 10 days from onset of symptoms [10] at which point infectious virus may no longer be found despite ongoing detection of viral load by RT-PCR [9, 11]. Work by La Scola’s group [12, 13] conducted RT-PCR testing and virus culture on positive samples with known Ct values showing that virus could not be isolated from samples collected after day eight of symptom onset, despite ongoing high viral loads. Only 70% of 3790 positive samples with Ct < 25 could be cultured, compared with less than 3% of the cases with Ct values above 35. In agreement, Bullard et al., [14] took 90 SARS-CoV-2 RT-PCR–confirmed positive samples and demonstrated no viral growth in samples with a Ct > 24 or symptom onset to test time > 8 days suggesting the infectivity of patients with Ct > 24 and duration of symptoms > 8 days may be low. Current guidelines from the Centers for Disease Control and Prevention and World Health Organization also call for patients to isolate for 10 days after onset of symptoms, recognising that individuals are not likely to be infectious after that period. For these reasons (and assuming good sampling and sample handling) use of an assay of lower analytical sensitivity (or application of a cut-off in an assay of higher analytical sensitivity) may be preferable for an asymptomatic screening programme designed to detect potentially infectious individuals, to avoid ‘false-positive’ detection of residual RNA in individuals who have recovered from COVID-19.

On the basis of these findings and in-line with best documented practices at the time, implementation of a University of Leicester Asymptomatic COVID-19 RT-LAMP Screening Programme followed a swab based RNA-extraction with rapid RT-LAMP assay targeting the Orf1a with a reaction TTP cut off of 20 minutes, equivalent to Ct < 30. Importantly, the cost per test of this assay was about half that of standard RT-PCR, widely in use for COVID-19 diagnostics. In any asymptomatic population when the infection prevalence is low, test specificity must be sufficiently high to ensure an acceptable positive predictive value [15]. In contrast to other RT-LAMP COVID-19 diagnostic workflows, an internal total RNA control reaction was included for each sample, minimizing the chance of false negative reporting and consequently improving the negative predictive value of this assay. Carry-over contamination prevention reagents (dUTP and UDG from NEB) were also included as standard, minimizing a serious and known challenge associated with isothermal amplification strategies. Targeting of the genomic sequence, combined with product melt curve specify check and confirmation by NHS laboratory RT-PCR found a SARS-CoV-2 asymptomatic infection incidence of 0.54% within this population. Given that at least 50% of new SARS-CoV-2 infections are estimated to originate from exposure to asymptomatic individuals [16] able to transmit the virus for an extended period [17] (perhaps longer than 14 days), this value is relatively low, suggesting that social distancing behaviors and the prolonged restrictions imposed on this population have been successful. Due to the potential of significant silent spread by asymptomatic persons [18–20] it is crucial that screening efforts such as the work described here are directed at those without symptoms in a targeted manner. In conclusion, this work demonstrates the utility of a rapid and cost effective RT-LAMP assay for collapsing transmission of SARS-CoV-2 in an asymptomatic screening population.

## Supporting information

S1 Table RT-LAMP primer sequences targeting the large open reading frame. ORF1a-HMSe designed by Rabe and Cepko 2020, and the nucleocapsid, envelope and human beta-actin gene designed by NEB (Tanner *at al*., 2020 & Zhang 2020).
(PDF)

S2 Table Limit of detection of colorimetric end-point RT-LAMP reactions targeting genomic and sub-genomic regions of SARS-CoV-2. (PDF)

S3 Table RT-LAMP TTP values (minutes) for fluorescent endpoint assays targeting the Orf1a and dual N+E gene regions against 114 UHL confirmed positive and 88 UHL confirmed negative samples using the ABI StepOnePlus PCR platform. (PDF)

S4 Table RT-LAMP TTP values (minutes) for fluorescent endpoint assays targeting the Orf1a and dual N+E gene regions against 38 UHL confirmed positive and 41 UHL confirmed negative samples using the Qiagen Rotor-Gene Q platform. (PDF)

S5 Table RT-LAMP TTP values (minutes) for colorimetric endpoint assays targeting the Orf1a and dual N+E gene regions against 21 UHL confirmed positive and 40 UHL confirmed negative samples using the ABI StepOnePlus PCR platform. (PDF)

S6 Table RT-LAMP TTP values (minutes) for colorimetric endpoint triple target (Orf1a/N/E) assay against 41 UHL confirmed positive and 40 UHL confirmed negative samples using the ABI StepOnePlus PCR platform. (PDF)

## Abbreviations

SARS-CoV-2: Severe acute respiratory syndrome coronavirus 2
RT-LAMP: reverse transcriptase loop-mediated isothermal amplification
RT-PCR: reverse transcriptase polymerase chain reaction
DSe: Diagnostic sensitivity
DSp: Diagnostic specificity
