# Supplementary material for: A rapid RT-LAMP SARS-CoV-2 screening assay for collapsing asymptomatic COVID-19 transmission
Source: PLoS One. 2022 Sep 1;17(9):e0273912. doi: 10.1371/journal.pone.0273912 (PMC9436079; doi:10.1371/journal.pone.0273912)
Supplement: S1 Table — ORF1a-HMSe designed by Rabe and Cepko 2020, and the nucleocapsid, envelope and human beta-actin gene designed by NEB (Tanner at al., 2020 & Zhang 2020). (PDF) [file pone.0273912.s001.pdf]

S1 Table. RT-LAMP primer sequences. ORF1a-HMSe designed by Rabe and Cepko 2020, the nucleocapsid, envelope and human beta-actin gene designed by NEB (Tanner *at al.,* 2020 & Zhang 2020).

**Primer ID:**	**Primer Sequence:**
ORF1a HMSe F3	CGGTGGACAAATTGTCAC
ORF1a HMSe B3	CTTCTCTGGATTTAACACACTT
ORF1a HMSe FIP	TCAGCACACAAAGCCAAAAATTTATTTTTCTGTGCAAAGGAAATTAAGGAG
ORF1a HMSe BIP	TATTGGTGGAGCTAAACTTAAAGCCTTTTCTGTACAATCCCTTTGAGTG
ORF1a HMSe LF	TTACAAGCTTAAAGAATGTCTGAACACT
ORF1a HMSe LB	TTGAATTTAGGTGAAACATTTGTCACG
E-F3	TGAGTACGAACTTATGTACTCAT
E-B3	TTCAGATTTTTAACACGAGAGT
E-FIP	ACCACGAAAGCAAGAAAAAGAAGTTCGTTTCGGAAGAGACAG
E-BIP	TTGCTAGTTACACTAGCCATCCTTAGGTTTTACAAGACTCACGT
E-LB	GCGCTTCGATTGTGTGCGT
E-LF	CGCTATTAACTATTAACG
N-F3	ACCAGGAACTAATCAGACAAG
N-B3	GACTTGATCTTTGAAATTTGGATCT
N-FIP	TTCCGAAGAACGCTGAAGCGGAACTGATTACAAACATTGGCC
N-BIP	CGCATTGGCATGGAAGTCACAATTTGATGGCACCTGTGTA
N-LB	GGGGGCAAATTGTGCAATTTG
N-LF	CTTCGGGAACGTGGTTGACC
ACTB F3	AGTACCCCATCGAGCACG
ACTB B3	AGCCTGGATAGCAACGTACA
ACTB FIP	GAGCCACACGCAGCTCATTGTATCACCAACTGGGACGACA
ACTB BIP	CTGAACCCCAAGGCCAACCGGCTGGGGTGTTGAAGGTC
ACTB LF	TGTGGTGCCAGATTTTCTCCA
ACTB LB	CGAGAAGATGACCCAGATCATGT
