# Supplementary material for: A rapid RT-LAMP SARS-CoV-2 screening assay for collapsing asymptomatic COVID-19 transmission
Source: PLoS One. 2022 Sep 1;17(9):e0273912. doi: 10.1371/journal.pone.0273912 (PMC9436079; doi:10.1371/journal.pone.0273912)
Supplement: S2 Table — (PDF) [file pone.0273912.s002.pdf]

S2 Table. Limit of detection of colorimetric end-point RT-LAMP reactions targeting genomic and sub-genomic regions of SARS-CoV-2. Twist Bioscience synthetic positive control RNA (control 2 GenBank ID MN908947.3, GISAID Wuhan-Hu-1) was serially diluted to 10,000, 1,000, 500, 100, 50 and 10 copies of viral sequence per reaction. Water no template control (NTC) were included in each reaction. Reactions were performed at 65°C for 40 minutes on the ABI StepOnePlus PCR platform. Representative colorimetric reactions for each primer set are shown. Data summary is an average of 3 independent experiments, performed in duplicate and presented as mean TTP ± S.E.M.

Copies / reaction	Colorimetric RT-LAMP Mean TTP (min) ± SEM (N)		
	**Orf1a**	**N+E**	**Orf1a+N+E**
**10,000**	8.2 ± 0.16 (8/8)	13.2 ± 0.48 (8/8)	10.0 ± 0.28 (8/8)
**1,000**	10.0 ± 0.22 (8/8)	16.0 ± 0.78 (8/8)	12.0 ± 0.35 (8/8)
**500**	10.7 ± 0.35 (8/8)	15.8 ± 0.78 (8/8)	12.7 ± 0.28 (8/8)
**100**	12.4 ± 0.64 (3/8)	19.4 ± 1.23 (7/8)	16.2 ± 1.72 (5/8)
**50**	13.4 ± 0.94 (4/8)	24.5 ± 1.42 (4/8)	20.2 ± 2.15 (5/8)
**10**	na	na	22.5 ± 7.75 (2/8)
**NTC**	na	na	na

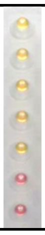
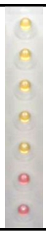
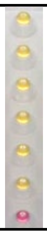

Yellow = amplification
Pink = no amplification ('na')
