# Supplementary material for: A rapid RT-LAMP SARS-CoV-2 screening assay for collapsing asymptomatic COVID-19 transmission
Source: PLoS One. 2022 Sep 1;17(9):e0273912. doi: 10.1371/journal.pone.0273912 (PMC9436079; doi:10.1371/journal.pone.0273912)
Supplement: S3 Table — (PDF) [file pone.0273912.s003.pdf]

S3 Table. RT-LAMP TTP values (minutes) for fluorescent endpoint assays targeting the Orf1a and dual N+E gene regions against 114 UHL confirmed positive and 88 UHL confirmed negative samples using the ABI StepOnePlus PCR platform run for 40 minutes. RT-PCR samples were considered positive when either the E or S gene amplify upder Ct of 40.

Sample ID	RT-PCR status:	RT-PCR Ct:		RT-LAMP TTP (min)		
		Altona E	Altona S	ORF1a	N+E gene	RNA control
1	Pos	11.1	8.4	8.0	8.3	11.5
2	Pos	12.2	10.5	8.9	8.2	18.4
3	Pos	12.5	11.3	10.1	8.5	11.7
4	Pos	12.6	11.6	11.8	10.7	11.9
5	Pos	14.8	13.7	10.8	9.5	10.9
6	Pos	15.1	13.8	11.2	9.5	11.7
7	Pos	15.1	14.0	9.8	9.9	11.8
8	Pos	15.2	14.1	11.0	9.6	10.8
9	Pos	15.2	14.2	11.5	9.3	10.7
10	Pos	15.3	14.3	10.3	11.0	12.8
11	Pos	15.4	14.3	10.0	10.2	11.4
12	Pos	16.2	14.7	13.9	12.5	11.9
13	Pos	16.3	15.3	11.9	9.8	10.1
14	Pos	16.4	15.4	11.9	10.0	8.6
15	Pos	16.6	15.7	11.1	10.4	13.0
16	Pos	16.6	15.0	11.6	12.9	19.4
17	Pos	16.8	14.9	18.3	20.4	18.5
18	Pos	17.0	15.7	9.8	9.7	11.8
19	Pos	17.2	15.9	12.4	10.6	10.0
20	Pos	17.2	16.1	10.7	11.5	13.7
21	Pos	17.4	16.3	10.9	11.8	12.5
22	Pos	17.7	17.5	13.9	16.4	13.4
23	Pos	17.7	15.7	11.1	13.3	17.8
24	Pos	18.0	15.9	11.0	11.7	12.7
25	Pos	18.0	16.8	10.4	10.4	10.7
26	Pos	18.3	17.1	10.6	10.7	12.0
27	Pos	18.7	18.0	10.6	11.1	11.0
28	Pos	18.7	18.1	11.6	13.6	12.9
29	Pos	18.8	16.5	14.2	13.4	11.8
30	Pos	18.8	17.9	10.7	10.5	8.8
31	Pos	19.1	17.9	13.7	11.8	10.4
32	Pos	19.1	17.6	13.2	11.3	11.2
33	Pos	19.7	18.9	10.9	11.1	12.5
34	Pos	19.8	17.9	12.4	15.0	20.7
35	Pos	19.9	19.3	11.1	10.9	10.5
36	Pos	20.2	19.1	12.0	12.1	11.7
37	Pos	20.3	19.3	12.0	12.2	11.5
38	Pos	20.4	17.6	12.4	13.8	13.5
39	Pos	20.4	19.1	11.7	11.3	12.7
40	Pos	20.5	17.7	12.9	14.0	12.5
41	Pos	20.6	19.3	11.7	11.7	10.7
42	Pos	21.1	19.9	11.7	11.9	11.9
43	Pos	21.3	20.0	14.6	12.6	11.9
44	Pos	21.3	19.6	13.4	16.0	16.2
45	Pos	21.8	19.9	13.6	15.7	12.3
46	Pos	22.3	20.2	13.6	15.6	11.8
47	Pos	22.4	21.3	13.3	13.6	26.7
48	Pos	22.5	21.5	12.0	12.3	12.2
49	Pos	22.8	22.1	14.0	14.8	10.9
50	Pos	22.8	22.0	12.8	11.9	10.7
51	Pos	23.1	22.0	13.1	14.8	17.8

52	Pos	23.2	20.5	13.7	15.6	11.9
53	Pos	23.3	20.8	14.8	16.4	16.3
54	Pos	23.3	20.6	17.1	15.5	13.1
55	Pos	23.3	22.2	12.5	12.2	11.3
56	Pos	23.4	20.8	13.9	14.7	12.1
57	Pos	23.4	21.0	15.2	16.0	16.9
58	Pos	23.4	22.6	16.6	13.4	11.8
59	Pos	23.6	24.6	13.0	12.3	10.6
60	Pos	24.0	22.8	na	na	10.9
61	Pos	24.1	23.1	15.7	16.1	14.9
62	Pos	24.1	23.0	16.1	13.9	12.6
63	Pos	24.3	24.3	16.7	13.9	10.7
64	Pos	24.5	23.0	14.3	15.1	13.9
65	Pos	24.5	23.4	16.0	15.2	10.9
66	Pos	24.8	23.8	12.9	13.7	13.6
67	Pos	24.8	22.2	14.7	18.2	11.7
68	Pos	24.9	22.5	17.6	17.2	12.7
69	Pos	25.0	24.2	16.1	15.1	11.8
70	Pos	25.1	23.9	13.5	14.8	11.1
71	Pos	25.1	24.1	na	na	13.7
72	Pos	25.2	21.8	13.9	13.1	11.5
73	Pos	25.2	24.2	13.0	13.8	16.0
74	Pos	25.6	22.9	14.4	15.4	13.8
75	Pos	25.9	25.0	17.5	17.2	11.8
76	Pos	26.1	23.9	15.2	16.3	11.5
77	Pos	26.7	23.8	16.7	23.1	15.9
78	Pos	27.1	26.4	14.8	14.7	12.8
79	Pos	27.1	25.3	21.2	22.8	16.8
80	Pos	27.2	24.8	15.4	15.6	11.8
81	Pos	27.4	24.8	16.0	24.9	13.5
82	Pos	27.5	24.6	15.9	29.2	11.1
83	Pos	27.6	27.3	15.2	17.5	10.4
84	Pos	27.6	26.8	na	24.6	12.0
85	Pos	28.1	27.2	17.3	26.6	15.0
86	Pos	28.3	27.2	na	15.2	9.7
87	Pos	28.6	25.8	15.9	na	11.0
88	Pos	28.7	26.0	26.1	31.1	12.6
89	Pos	28.9	25.6	21.9	16.7	15.1
90	Pos	29.1	28.3	17.4	na	12.5
91	Pos	29.2	26.2	na	19.5	11.4
92	Pos	29.9	28.3	na	43.3	12.2
93	Pos	29.9	29.2	14.5	18.0	12.1
94	Pos	30.4	27.2	22.8	na	17.6
95	Pos	30.4	30.0	na	na	11.6
96	Pos	30.6	30.1	na	31.5	14.6
97	Pos	30.6	44.6	15.1	na	9.6
98	Pos	30.8	32.1	14.6	18.2	10.9
99	Pos	30.8	30.7	16.9	41.7	12.0
100	Pos	31.8	31.8	na	26.0	10.3
101	Pos	32.2	29.9	na	na	14.7
102	Pos	32.2	30.8	na	na	11.7
103	Pos	32.5	30.5	na	na	15.1
104	Pos	32.6	29.6	na	na	12.7
105	Pos	33.0	30.8	na	na	11.7
106	Pos	33.1	32.0	na	na	14.3
107	Pos	33.2	31.4	na	na	12.9
108	Pos	33.3	33.3	na	na	13.7
109	Pos	35.6	34.6	na	na	24.6

110	Pos	39.5	38.2	na	na	14.3
111	Pos	na	32.7	na	na	14.1
112	Pos	na	33.2	na	na	20.2
113	Pos	na	33.2	na	na	16.9
114	Pos	na	33.3	na	na	14.8
1	Neg	na	na	na	na	15.7
2	Neg	na	na	na	na	15.5
3	Neg	na	na	na	na	15.9
4	Neg	na	na	na	na	15.8
5	Neg	na	na	na	na	15.8
6	Neg	na	na	na	na	17.1
7	Neg	na	na	na	na	16.6
8	Neg	na	na	na	na	16.5
9	Neg	na	na	na	na	16.3
10	Neg	na	na	na	na	21.8
11	Neg	na	na	na	na	22.4
12	Neg	na	na	na	na	17.1
13	Neg	na	na	na	na	18.0
14	Neg	na	na	na	na	15.3
15	Neg	na	na	na	na	17.6
16	Neg	na	na	na	na	19.7
17	Neg	na	na	na	na	21.1
18	Neg	na	na	na	na	16.1
19	Neg	na	na	na	na	16.7
20	Neg	na	na	na	na	15.8
21	Neg	na	na	na	na	19.3
22	Neg	na	na	na	na	18.7
23	Neg	na	na	na	na	19.9
24	Neg	na	na	na	na	19.9
25	Neg	na	na	na	na	18.2
26	Neg	na	na	na	38.0	20.9
27	Neg	na	na	na	na	19.6
28	Neg	na	na	na	36.9	19.2
29	Neg	na	na	na	37.3	18.5
30	Neg	na	na	na	na	14.8
31	Neg	na	na	na	na	21.3
32	Neg	na	na	na	na	14.9
33	Neg	na	na	na	na	16.5
34	Neg	na	na	na	na	15.8
35	Neg	na	na	na	40.9	18.9
36	Neg	na	na	na	na	19.9
37	Neg	na	na	na	na	17.6
38	Neg	na	na	na	na	17.9
39	Neg	na	na	na	na	17.6
40	Neg	na	na	na	na	17.9
41	Neg	na	na	na	na	18.9
42	Neg	na	na	na	na	19.3
43	Neg	na	na	na	na	19.1
44	Neg	na	na	na	na	17.4
45	Neg	na	na	na	na	14.1
46	Neg	na	na	na	na	18.2
47	Neg	na	na	na	na	13.8
48	Neg	na	na	na	na	15.9
49	Neg	na	na	na	na	14.9
50	Neg	na	na	na	na	15.2
51	Neg	na	na	na	na	15.1
52	Neg	na	na	na	na	15.7
53	Neg	na	na	na	na	16.1

54	Neg	na	na	na	na	16.7
55	Neg	na	na	na	na	19.2
56	Neg	na	na	na	na	13.6
57	Neg	na	na	na	na	20.3
58	Neg	na	na	na	na	13.8
59	Neg	na	na	na	na	15.5
60	Neg	na	na	na	na	15.5
61	Neg	na	na	na	na	15.4
62	Neg	na	na	na	na	17.4
63	Neg	na	na	na	na	15.8
64	Neg	na	na	na	na	21.4
65	Neg	na	na	na	na	16.7
66	Neg	na	na	na	na	16.2
67	Neg	na	na	na	na	14.1
68	Neg	na	na	na	na	16.6
69	Neg	na	na	na	na	13.9
70	Neg	na	na	na	na	16.5
71	Neg	na	na	na	na	14.9
72	Neg	na	na	na	na	16.2
73	Neg	na	na	na	na	17.0
74	Neg	na	na	na	na	16.7
75	Neg	na	na	na	na	19.9
76	Neg	na	na	na	41.7	19.3
77	Neg	na	na	na	na	28.0
78	Neg	na	na	na	na	19.2
79	Neg	na	na	na	na	16.4
80	Neg	na	na	na	na	26.0
81	Neg	na	na	na	na	19.7
82	Neg	na	na	na	42.7	16.2
83	Neg	na	na	na	na	17.8
84	Neg	na	na	na	na	18.0
85	Neg	na	na	na	na	19.5
86	Neg	na	na	na	na	17.0
87	Neg	na	na	na	na	17.5
88	Neg	na	na	na	44.7	17.8

na = no amplification.
Data in grey box = negative swab samples
