# Supplementary material for: A rapid RT-LAMP SARS-CoV-2 screening assay for collapsing asymptomatic COVID-19 transmission
Source: PLoS One. 2022 Sep 1;17(9):e0273912. doi: 10.1371/journal.pone.0273912 (PMC9436079; doi:10.1371/journal.pone.0273912)
Supplement: S5 Table — (PDF) [file pone.0273912.s005.pdf]

S5 Table. Colorimetric endpoint assay. 30 minute TTP cut off. Amplfiication after which time is deemed non-specific.

Sample ID	RT-PCR status:	RT-PCR Ct:		RT-LAMP TTP (min)		
		Altona E	Altona S	ORF1a	N+E gene	RNA control
1	Pos	14.8	13.7	10.8	9.5	10.9
2	Pos	16.6	15.0	11.6	12.9	19.4
3	Pos	18.0	15.9	11.0	11.7	12.7
4	Pos	20.4	17.6	12.4	13.8	13.5
5	Pos	20.5	17.7	12.9	14.0	12.5
6	Pos	21.3	19.6	13.4	16.0	16.2
7	Pos	22.3	20.2	13.6	15.6	11.8
8	Pos	22.8	22.0	12.8	11.9	10.7
9	Pos	23.4	21.0	15.2	16.0	16.9
10	Pos	24.9	22.5	17.6	17.2	12.7
11	Pos	25.2	24.2	13.0	13.8	16.0
12	Pos	25.6	22.9	14.4	15.4	13.8
13	Pos	26.7	23.8	16.7	23.1	15.9
14	Pos	27.1	25.3	21.2	22.8	16.8
15	Pos	27.4	24.8	16.0	24.9	13.5
16	Pos	28.6	25.8	15.9	na	11.0
17	Pos	28.9	25.6	21.9	16.7	15.1
18	Pos	30.6	29.5	na	31.5	14.6
19	Pos	32.2	29.9	na	na	14.7
20	Pos	33.2	31.4	na	na	12.9
21	Pos	39.5	38.2	na	na	14.3
1	Neg	na	na	na	na	13.7
2	Neg	na	na	na	na	12.3
3	Neg	na	na	na	na	11.3
4	Neg	na	na	na	na	13.3
5	Neg	na	na	na	na	11.3
6	Neg	na	na	na	na	9.6
7	Neg	na	na	na	na	10.0
8	Neg	na	na	na	na	9.6
9	Neg	na	na	na	na	11.1
10	Neg	na	na	na	na	12.7
11	Neg	na	na	na	na	10.6
12	Neg	na	na	na	na	10.3
13	Neg	na	na	na	na	10.7
14	Neg	na	na	na	na	11.1
15	Neg	na	na	na	na	11.6
16	Neg	na	na	na	na	12.4
17	Neg	na	na	na	na	11.8
18	Neg	na	na	na	na	13.7
19	Neg	na	na	na	na	10.4
20	Neg	na	na	na	na	13.7
21	Neg	na	na	na	na	9.8
22	Neg	na	na	na	na	11.4
23	Neg	na	na	na	na	10.4
24	Neg	na	na	na	na	10.8
25	Neg	na	na	na	na	11.9
26	Neg	na	na	na	na	12.5
27	Neg	na	na	na	na	14.7
28	Neg	na	na	na	na	15.3
29	Neg	na	na	na	na	9.8
30	Neg	na	na	na	na	14.3
31	Neg	na	na	na	na	11.6
32	Neg	na	na	na	na	11.8

33	Neg	na	na	na	na	10.4
34	Neg	na	na	na	na	12.9
35	Neg	na	na	na	na	11.7
36	Neg	na	na	na	na	14.6
37	Neg	na	na	na	na	10.4
38	Neg	na	na	na	na	8.9
39	Neg	na	na	na	na	10.9
40	Neg	na	na	na	na	9.0

na = no amplification.
Data in grey box = negative swab samples
