# Supplementary material for: A rapid RT-LAMP SARS-CoV-2 screening assay for collapsing asymptomatic COVID-19 transmission
Source: PLoS One. 2022 Sep 1;17(9):e0273912. doi: 10.1371/journal.pone.0273912 (PMC9436079; doi:10.1371/journal.pone.0273912)
Supplement: S6 Table — (PDF) [file pone.0273912.s006.pdf]

S6 Table. RT-LAMP TTP values (minutes) for colorimetric endpoint triple target (Orf1a/N/E) assay against 41 UHL confirmed positive and 40 UHL confirmed negative samples using the ABI StepOnePlus PCR platform.

Sample ID	RT-PCR Ct:		TTP (min)
	Altona E	Altona S	Orf1a/N/E
1	12.2	10.5	5.8
2	14.8	13.6	6.8
3	15.1	14.0	7.0
4	15.9	14.3	7.4
5	17.4	16.3	8.2
6	17.4	15.9	8.0
7	18.1	16.9	8.1
8	18.9	18.0	8.1
9	20.1	28.4	13.0
10	20.2	19.0	8.8
11	20.3	19.3	8.7
12	20.4	19.0	8.8
13	21.2	20.3	8.9
14	22.1	21.1	9.2
15	22.2	21.2	9.3
16	22.4	21.3	9.6
17	23.1	22.0	9.7
18	23.2	21.8	10.0
19	23.8	22.9	10.3
20	24.1	23.1	9.9
21	24.4	23.2	10.2
22	24.5	23.0	10.2
23	24.8	24.0	10.8
24	25.0	24.1	10.5
25	25.6	24.3	10.8
26	25.7	24.5	10.7
27	25.9	25.0	10.7
28	26.7	25.6	12.6
29	26.8	25.2	11.3
30	27.0	26.1	12.7
31	28.1	27.2	11.7
32	29.9	28.3	19.6
33	30.4	29.3	12.6
34	30.8	30.1	na
35	31.1	29.4	15.6
36	31.2	30.3	16.4
37	32.5	32.0	19.1
38	33.1	34.2	13.0
39	34.1	32.5	18.7
40	35.6	34.6	na
41	37.4	35.0	na
1	na	na	13.7
2	na	na	12.3
3	na	na	11.3

4	na	na	13.3
5	na	na	11.3
6	na	na	9.6
7	na	na	10.0
8	na	na	9.6
9	na	na	11.1
10	na	na	12.7
11	na	na	10.6
12	na	na	10.3
13	na	na	10.7
14	na	na	11.1
15	na	na	11.6
16	na	na	12.4
17	na	na	11.8
18	na	na	13.7
19	na	na	10.4
20	na	na	13.7
21	na	na	9.8
22	na	na	11.4
23	na	na	10.4
24	na	na	10.8
25	na	na	11.9
26	na	na	12.5
27	na	na	14.7
28	na	na	15.3
29	na	na	9.8
30	na	na	14.3
31	na	na	11.6
32	na	na	11.8
33	na	na	10.4
34	na	na	12.9
35	na	na	11.7
36	na	na	14.6
37	na	na	10.4
38	na	na	8.9
39	na	na	10.9
40	na	na	9.0

Na = no amplification

Data in grey box = negative swab samples
